# Association of embolization with long-term outcomes in brain arteriovenous malformations: a propensity score-matched analysis using nationwide multicenter prospective registry data

**DOI:** 10.1097/JS9.0000000000000341

**Published:** 2023-05-24

**Authors:** Yu Chen, Heze Han, Hengwei Jin, Xiangyu Meng, Li Ma, Ruinan Li, Zhipeng Li, Debin Yan, Haibin Zhang, Kexin Yuan, Ke Wang, Yang Zhao, Yukun Zhang, Weitao Jin, Runting Li, Fa Lin, Qiang Hao, Hao Wang, Xun Ye, Shuai Kang, Dezhi Gao, Jun Pu, Zhiyong Shi, Xiaofeng Chao, Zhengfeng Lin, Junlin Lu, Jiangan Li, Shibin Sun, Ali Liu, Xiaolin Chen, Youxiang Li, Yuanli Zhao, Shuo Wang

**Affiliations:** a Department of Neurosurgery; b Department of Interventional Neuroradiology; c Department of Gamma-Knife center, Beijing Tiantan Hospital, Capital Medical University; d China National Clinical Research Center for Neurological Diseases; e Department of Neurosurgery, Peking University International Hospital, Peking University, Beijing; f Department of Neurosurgery, The First Hospital of Hebei Medical University, Hebei Medical University, Shijiazhuang; g Department of Neurosurgery, Shanxi Provincial People’s Hospital, Shanxi; h Department of Neurosurgery, The Affiliated Wuxi NO.2 People’s Hospital of Jiangnan University, Wuxi; i Department of Neurosurgery, Nanjing Drum Tower Hospital, Affiliated to Nanjing University, Nanjing; j Department of Neurosurgery, The Second Affiliated Hospital of Xuzhou Medical University, Jiangsu; k Department of Neurosurgery, The First People’s Hospital of Qinzhou, Guangxi; l Department of Neurosurgery, West China Hospital, Sichuan University, Chengdu, Sichuan; m First Department of Neurosurgery, The Second Affiliated Hospital of Kunming Medical University, Kunming, China

**Keywords:** arteriovenous malformation, conservative management, death, embolization, hemorrhagic stroke, neurological outcomes

## Abstract

Brain arteriovenous malformations (AVMs) account for 25% of hemorrhagic strokes in young adults. Although embolization has been widely performed as a stand-alone procedure to cure brain AVM, it is undermined whether patients benefit from this treatment. This study aimed to compare the long-term outcome of hemorrhagic stroke or death in patients with either conservative management or stand-alone embolization for AVM.

**Methods:**

The study population was derived from a nationwide multicenter prospective collaboration registry (the MATCH registry) between August 2011 and August 2021. The propensity score-matched survival analysis was performed in the overall and stratified AVM cases (unruptured and ruptured), respectively, to compare the long-term outcome of hemorrhagic stroke or death, and neurological status. The efficacy of distinct embolization strategies was also evaluated. Hazard ratios (HRs) with 95% CI were calculated using Fine-Gray competing risk models.

**Results:**

Of the 3682 consecutive AVMs, 906 underwent either conservative management or embolization as the stand-alone management strategy. After propensity score matching, a total of 622 (311 pairs) patients constituted an overall cohort. The unruptured and ruptured subgroups were composed of 288 cases (144 pairs) and 252 cases (126 pairs), respectively. In the overall cohort, embolization did not prevent long-term hemorrhagic stroke or death compared with conservative management [2.07 vs. 1.57 per 100 patient-years; HR, 1.28 (95% CI, 0.81–2.04)]. Similar results were maintained in both unruptured AVMs [1.97 vs. 0.93 per 100 patient-years; HR, 2.09 (95% CI, 0.99–4.41)] and ruptured AVMs [2.36 vs. 2.57 per 100 patient-years; HR, 0.76 (95% CI, 0.39–1.48)]. Stratified analysis showed that the target embolization might be beneficial for unruptured AVMs [HR, 0.42 (95% CI, 0.08–2.29)], while the curative embolization improved the outcome of ruptured AVMs [HR, 0.29 (95% CI, 0.10-0.87)]. The long-term neurological status was similar between these two strategies.

**Conclusions:**

This prospective cohort study did not support a substantial superiority of embolization over conservative management for AVMs in preventing long-term hemorrhagic stroke or death.

## Introduction

Highlight

- Embolization has no substantial significant superiority over conservative management for brain arteriovenous malformations in preventing long-term hemorrhagic stroke or death.

Brain arteriovenous malformations (AVMs) are tangles of abnormally dilated vessels without intervening capillaries, representing high-flow and low-resistance hemodynamic features due to direct arteriovenous shunting, with an estimated prevalence of ~50 cases per 100 000^1^. Hemorrhage is widely recognized as the major cause of morbidity and mortality for AVMs, and hemorrhage caused by AVMs accounts for ~25% of hemorrhagic strokes in young adults^2^. Consequently, the estimated risk of cumulative lifetime hemorrhage versus intervention must be weighed primarily in tailoring the therapeutic strategy^3^.

Endovascular embolization was typically performed as an adjunct to microsurgery or stereotactic radiosurgery (SRS) for AVMs to facilitate safer dissection of the nidus in microsurgery or reduce the nidus volume to improve the efficacy of SRS^4,5^. A growing body of literature has recently discussed curative embolization as a stand-alone treatment in selected lesions, with the advantages of minimal invasiveness and less procedures^4,6–8^. However, although many neurointerventionalists reported that the stand-alone embolization strategy could effectively prevent subsequent hemorrhage, especially the targeted embolization strategy^7,9–11^, all the latest clinical management guidelines for AVMs have not recommended the curative intent embolization strategy^12–14^.

The benefit of stand-alone embolization in ruptured/unruptured AVMs may be inconsistent^13^. In 2014, an international multicenter randomized controlled study, the ARUBA trial (A Randomized Trial of Unruptured Brain Arteriovenous Malformations), suggested that medical management was superior to intervention in preventing stroke and death in unruptured AVMs^15^. Moreover, they reported a perioperative stroke risk of up to 16% after the embolization procedure^6^.

Given the widespread use of stand-alone curative intent embolization in AVMs, its effectiveness profile should be assessed, and the beneficial effects should also be weighed against potential harms. This study aimed to compare the long-term outcomes for AVMs following conservative management and stand-alone embolization. This prospective cohort study compared the long-term risk of hemorrhagic stroke or death, and the proportion of favorable neurological status of patients with conservative management or stand-alone embolization. The outcomes for unruptured and ruptured AVMs were further analyzed separately. The efficacy of different embolization degrees and embolization strategies were also evaluated in both cohorts. This study can clarify whether the straightforward embolization of the proposed minimal invasiveness could be really beneficial, and help in treatment decisions for AVMs.

## Methods

### Study design, setting, and data sources

This study was a prospective cohort study using nationwide multicenter registry data from the registry of multimodality treatment for brain AVMs in MATCH (MATCH registry) to compare the risk of hemorrhagic stroke or death after conservative management versus embolization for AVMs^16^. This study was conducted following the STROCSS reporting guidelines, Supplemental Digital Content 1, http://links.lww.com/JS9/A573.

The MATCH registry is a nationwide multicenter prospective registry to study the natural history of AVMs in the MATCH population and to explore the optimal individualized treatment strategy for AVMs (ClinicalTrials.gov register, NCT 04572568). A comprehensive protocol for data quality management in the MATCH registry was demonstrated in Supplemental Methods, Supplemental Digital Content 2, http://links.lww.com/JS9/A574. Previous studies have demonstrated the database’s validity and quality for research^5,17^. This study was carried out according to the guideline of the 1964 Helsinki Declaration and was approved by the institutional ethics committee (IRB approval number: KY 2020-003-01). Written informed consent was obtained at admission before entering the study.

### Study population

All AVMs admitted at hospitals of the MATCH registry between 1 August 2011, and 1 August 2021, were reviewed to identify patients who underwent embolization procedures or ongoing conservative management. Patients with clinical baseline data missing, or microsurgical resection, or SRS treatment were excluded. Consistent with the ARUBA, conservative management was defined as receiving only therapeutic treatment without intervention in the focal structure, such as medication for existing medical disorders (e.g. seizures, headaches) or any coexisting vascular risk factors (diabetes, arterial hypertension) as needed. Patients who lost to follow-up were also excluded. Onyx (Covidien) was the most commonly used embolic agent in the MATCH Research Collaborative Unit.

Two independent sub-cohorts were defined according to AVM rupture or unruptured at initial presentation to investigate the effect of embolization in the sub-cohorts. In the stratified analysis, the embolization degree (minority: <50% of AVM lesion volume; majority: 50–99%; complete obliteration: 100%) and embolization strategy (target; palliative; curative) were determined by two credentialed senior endovascular specialists (MATCH and MATCH) according to the last embolization before the primary outcome occurred (patients with the primary outcome) or the last clinical follow-up (patients with no primary outcome). The extent of embolization was measured by the change of nidus volume, as was defined in some previous literatures^18^. For the embolization strategy, a target embolization was defined as embolizing high-risk angiographic features that predispose AVM to rupture, such as flow-related aneurysms, intranidal fistula, Hou *et al* ^19^. A palliative embolization aimed to block high-flow feeding arteries or the largest fistula, to relieve venous drainage hypertension, and thereby palliate symptoms^1^. A curative embolization was to obliterate feeding arteries as much as possible to achieve complete occlusion of the nidus^10^.

### Baseline characteristics

The study population was characterized considering the following potential confounders: demographic factors (age at diagnosis, sex), clinical presentations (hemorrhage, seizure, neurological deficit), modified Rankin Scale (mRS) at admission, morphological features (nidus location, ventricular system involvement, size, eloquent region), Spetzler-Martin (SM) grade, and angio-architectural parameters.

The definitions of angio-architectural parameters were consistent with the reported terminology provided by the joint committee led by the American Society of Interventional and Therapeutic Neuroradiology^20^, including feeding artery dilation, single feeder, multiple source supply, perforating artery, flow-related aneurysm, diffuse nidus, exclusive deep drainage, any deep drainage, single draining vein, vein drainage stenosis, and venous aneurysm. The morphological features and angio-architectural parameters were confirmed by two credentialed senior neuroradiologists (MATCH and MATCH).

### Outcomes and exposures

The primary outcome was the composite event of hemorrhagic stroke or death. Hemorrhagic stroke was defined as a symptomatic event (any new focal neurological deficit, seizure, or new-onset severe headache) with imaging findings (intracranial hematoma or subarachnoid hemorrhage) on computed tomography or MRI, that could be attributed to AVM). The secondary outcome was the neurological status at the last follow-up. An mRS score of less than two was defined as a favorable neurological status.

Follow-up through clinic visits or telemedicine was performed at 3 months, annually (1, 2, and 3 years), and every 5 years after the initial treatment decision. Suspected individuals with cerebrovascular events were recorded with emphasis at each clinic visit or telephone contact. The inception point of the follow-up for conservative management was the date of clinical onset that led to the diagnosis of AVM. The follow-up for the embolization group started from the date of the first embolization after the AVM diagnosis^21^. The endpoint was the date of the primary outcome or last follow-up for patients with or without outcome events. For the secondary outcome, the endpoint was the last follow-up.

### Controlling for confounding

To minimize the impacts of potential confounding and selection bias, propensity score matching (PSM) was used to compensate for group differences in baseline characteristics. A propensity score was calculated using logistic regression, and 1:1 patient matching was performed using the nearest-neighbor matching method without replacement. Baseline characteristics, including demographic factors, clinical presentation, morphological features, and angio-architectural parameters, were matched between the conservative management and embolization groups, in three separate cohorts of the overall, unruptured, and ruptured AVMs, respectively. Either unruptured or ruptured AVM matching was derived from the unruptured or ruptured cohort separately, rather than from the overall matched AVMs. A caliper radius equal to a SD of 0.1 was set to prevent poor matching. Covariate balance was assessed using the standardized mean difference (SMD). Acceptable matching was indicated by an SMD less than 0.1. Unmatched patients were excluded.

### Statistical analyses

Baseline characteristics before and after matching were compared among the conservative management and embolization groups. Missing values were imputed using binary logistic regression models. Categorical variables were presented as percentages and continuous variables as mean with SD or median with interquartile range (IQR). In baseline comparison, the two-tailed *t*-test or Mann–Whitney test was used for continuous variables, and the χ^2^ test or Fisher’s exact test was utilized for the categorical variables as appropriate.

After PSM, person-years of follow-up for the primary outcome were calculated from the date of diagnosis or intervention to the last follow-up or outcome occurrence. The Poisson rate test was performed to compare the annual risk of the primary outcome between conservative management and embolization groups. Absolute rate differences were calculated. The Cox proportional hazards model was used to assess the effect of conservative management or embolization on the primary outcome. The proportional hazards assumption was tested by visually examining the Schoenfeld residuals. Estimates of treatment effects from Cox models were presented as hazard ratios (HRs) with 95% CI with all these analyses considering competing risks (Fine-Gray subdistribution hazard model) except for the sensitivity analyses. The 30-year cumulative incidence of the primary outcome was captured using cumulative incidence function curves. A global log-rank test was used to test for any differences between conservative management and embolization. Further stratified analyses were performed in the embolization group considering the impact of embolization degree (minority: <50%; majority: ≥50%, and <100%; obliteration: 100%) and embolization strategy (target; palliative; curative) on prognosis. Absolute risk reduction and number needed to treat were further calculated for embolization strategies with statistically significant differences. For the secondary outcomes, AVM-unrelated deaths constituted the competing risk events, and such cases were defined as censored cases to circumvent an overestimation of mortality. Relative risk (RR) was used to calculate the difference in the secondary outcome. Sensitivity analyses were conducted to compare HRs in the univariable Cox proportional hazard model before and after PSM, and the Fine-Gray model after PSM^22^. Prespecified subgroup analyses were performed within the matched cohorts of unruptured and ruptured AVMs, respectively, by categorizing sex (female and male), age at diagnosis (<18 years or ≥18 years), SM grade (1–3 or 4–5), location (superficial location or deep location), and drainage (superficial drainage or deep drainage). Tests of interaction were also performed across the above categories. Furthermore, to estimate the influence of unmeasured confounders on the observed treatment-outcome association, the E-value (representing the minimum strength of uncontrolled confounders to explain away this association) was calculated^23^.

All statistical analyses were performed using R version 4.0.3 (R Foundation for Statistical Computing). *P* values were two-sided, and *P*<0.05 was considered statistically significant.

## Results

### Study population and baseline characteristics

**Figure 1:**
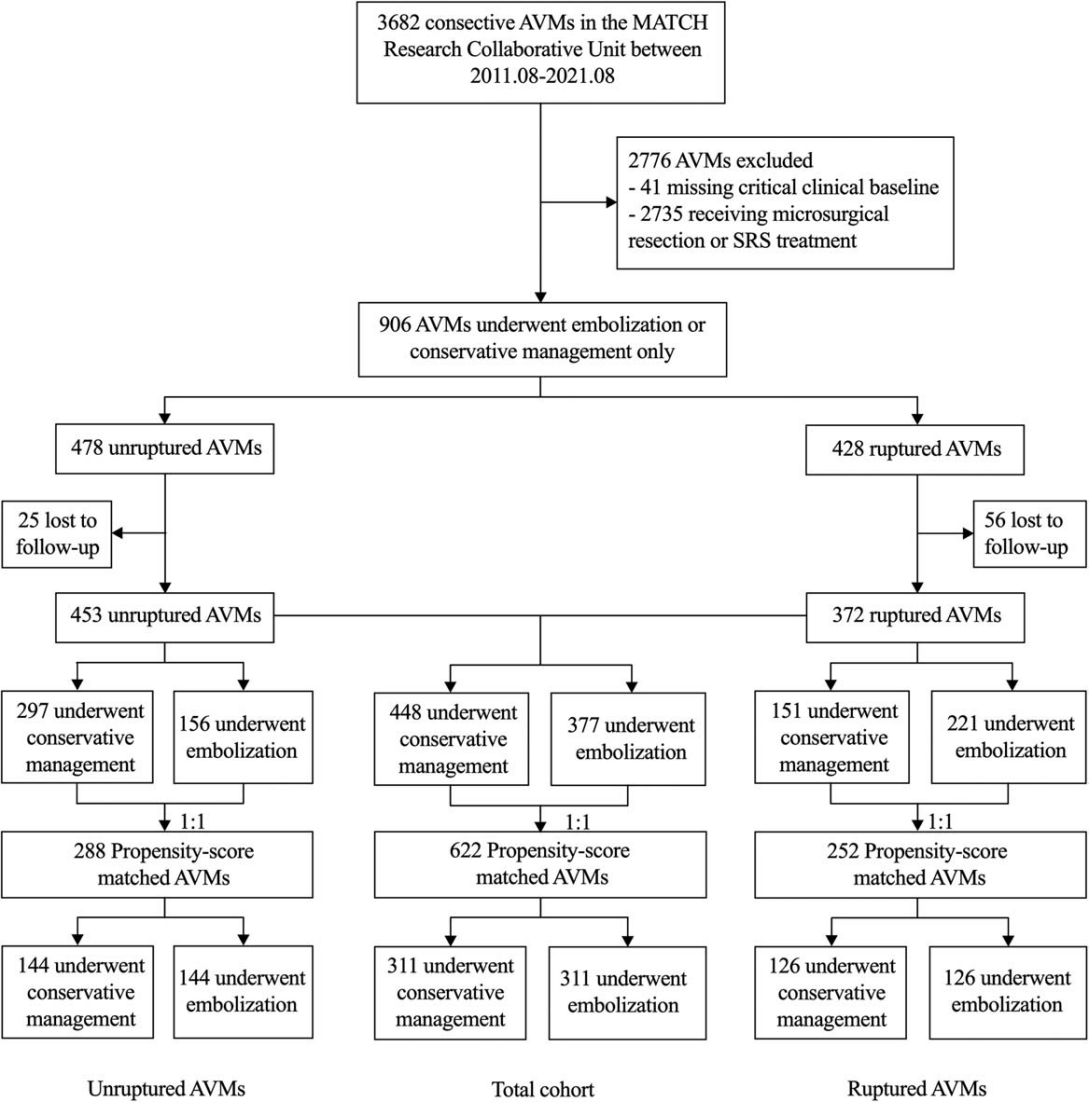
Flowchart of patient selection.

Of the 3682 consecutive AVMs from 10 participating sites in the MATCH Research Collaborative Unit covering eight provinces of MATCH between 1 August 2011, and 1 August 2021, a total of 906 AVMs underwent either embolization as the stand-alone management or conservative management. After excluding 81 AVMs (8.9%) who were lost to follow-up (Supplemental Table 1, Supplemental Digital Content 2, http://links.lww.com/JS9/A574), PSM was performed, yielding an overall cohort of 622 AVMs (311 per group with both unruptured and ruptured AVMs), an unruptured cohort of 288 AVM cases (144 per group), and a ruptured cohort of 252 cases (126 per group). Figure 1 shows the enrollment process.

Baseline characteristics before and after PSM were compared across the above cohorts in Supplemental Table 2, Supplemental Digital Content 2, http://links.lww.com/JS9/A574 and Supplemental Table 3, Supplemental Digital Content 2, http://links.lww.com/JS9/A574. After matching, the baseline differences were diminished between the two treatment groups, with no SMD exceeding 0.1 in each cohort (Supplemental Figure 1, Supplemental Digital Content 2, http://links.lww.com/JS9/A574). Most AVMs were of SM grade 1–3 (conservative management versus embolization: 73.0 versus 75.2% in the overall cohort; 69.4 versus 69.4% in the unruptured AVMs; 78.6 versus 81.0% in the ruptured AVMs). A total of 428 (overall, mean±SD: 1.4±0.7), 215 (unruptured, mean±SD: 1.5±0.9), and 163 (ruptured, mean±SD: 1.3±0.7) embolization procedures were performed in the three cohorts, respectively.

### Primary outcome

**Figure 2:**
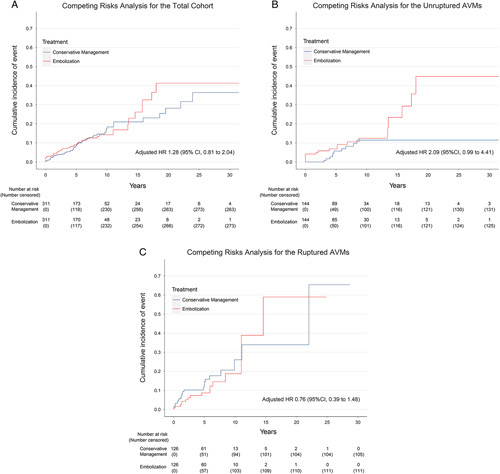
The cumulative incidence function curves of the primary outcome in the total cohort, unruptured AVMs, and ruptured AVMs.

In the overall cohort, the median follow-up for the primary outcome was 5.5 (IQR, 2.5 to 8.8) years. There was a trend toward a higher incidence of primary outcome in the embolization group than conservative management group [2.07 vs. 1.57, ARD, 0.50 (95% CI: −0.34–1.34) per 100 patient-years, *P*=0.244]. Embolization was shown to increase the risk of primary outcome by 28% [HR, 1.28 [95% CI: 0.81–2.04)] (Table 1). In the unruptured AVMs, the median follow-up for the primary outcome was 6.3 (IQR 3.0 to 9.8) years. The incidence of primary outcome was significantly higher in the embolization group [1.97 vs. 0.93, ARD, 1.04 (95% CI: 0.00–2.08) per 100 patient-years, *P*=0.046]. It should be noted that although the embolization group had a significantly higher risk of primary outcome before competing risks were taken into account [HR, 2.25 (95% CI: 1.04–4.85)], the difference was not observed in the subdistribution hazard model [HR, 2.09 (95% CI: 0.99–4.41)]. In the ruptured AVMs, the median follow-up duration for primary outcome was 4.8 (IQR 2.0 to 7.1) years. The incidence of primary outcome tended to be lower in the embolization group [2.36 vs. 2.57, ARD, -0.21 (95% CI: -1.81–1.39) per 100 patient-years, *P*=0.807]. And embolization was shown to decrease the risk of primary outcome by 24% [HR, 0.76 (95% CI: 0.39–1.48)]. Figure 2 showed the cumulative incidence function curves of primary outcome in the three matched cohorts. The E-value indicated that an unmeasured confounder required a strong association with embolization and primary outcome by an HR of 1.88-fold, 3.60-fold, 1.96-fold in the overall, unruptured and ruptured AVMs, respectively, to explain away the observed association.

**Table 1:** HRs, and 95% CIs for the primary outcome. All HRs use conservative management as the reference group.

	Conservative management		Embolization				Pre-PSM	PSM	PSM+CR	
	Number	Incidence rate per 100 patient-years	Number	Incidence rate per 100 patient-years	Rate difference (95% CI) per 100 person-years^a^	*P* ^b^	HR (95% CI)	HR (95% CI)	HR (95% CI)	E
Hemorrhagic stroke or death										
Total cohort	33	1.57	39	2.07	0.50 (−0.34–1.34)	0.244	1.15 (0.78–1.70)	1.31 (0.82–2.10)	1.28 (0.81–2.04)	1.88
Unruptured AVMs	11	0.93	19	1.97	1.04 (0.00–2.08)	0.046^b^	1.40 (0.78–2.52)	2.25 (1.04–4.85)^c^	2.09 (0.99–4.41)	3.60
Ruptured AVMs	21	2.57	15	2.36	−0.21 (−1.81–1.39)	0.807	0.73 (0.43–1.25)	0.76 (0.39–1.49)	0.76 (0.39–1.48)	1.96
Hemorrhagic stroke										
Total cohort	25	1.19	31	1.64	0.45 (−0.29–1.19)	0.235	1.17 (0.75–1.84)	1.40 (0.82–2.39)	1.36 (0.80–2.30)	2.06
Unruptured AVMs	6	0.51	15	1.55	1.04 (0.16–1.92)	0.018^c^	1.67 (0.84–3.30)	3.61 (1.31–9.99)^c^	3.59 (1.29–10.10)^c^	6.64
Ruptured AVMs	17	3.17	11	1.73	−1.44 (−3.24–0.36)	0.118	0.65 (0.35–1.17)	0.69 (0.32–1.49)	0.69 (0.32–1.48)	2.26
Death										
Total cohort	8	0.38	8	0.42	0.04 (−0.35– 0.43)	0.844	1.09 (0.49–2.43)	1.05 (0.39–2.79)	1.05 (0.40–2.79)	1.28
Unruptured AVMs	5	0.42	4	0.41	−0.01 (−0.55–0.53)	0.980	0.87 (0.27–2.84)	0.92 (0.25–3.43)	0.92 (0.24–3.48)	1.39
Ruptured AVMs	4	0.60	4	0.63	0.03 (−0.82–0.88)	0.946	1.21 (0.35–4.16)	1.06 (0.26–4.26)	1.06 (0.25–4.49)	1.31

a Positive differences indicate that the embolization group had a higher rate of outcome events than the conservative management group.

b The Poisson rate test was used to compare the annualized rate between groups.

c Statistically significant (*P*<0.05).

AVM, Arteriovenous Malformation; CR, Competing Risk; HR, Hazard Ratio; PSM, Propensity Score Match.

The primary outcome was further evaluated as hemorrhagic stroke and death, individually. In unruptured AVMs, the incidence of hemorrhagic stroke was significantly higher in the embolization group [1.55 vs. 0.51, ARD, 1.04 (95% CI: 0.16–1.92) per 100 patient-years, *P*=0.018]. Embolization was associated with a significantly higher risk of subsequent hemorrhage than conservative management [HR, 3.59 (95% CI: 1.29–10.10)]. Whereas in ruptured AVMs, embolization can reduce the risk of re-rupture by 31% [HR, 0.69 (95% CI: 0.32–1.48)]. In terms of mortality, there was no significant difference between the two treatment groups in any of the three matched cohorts.

### Stratified analysis of embolization degree and embolization strategy

**Figure 3:**
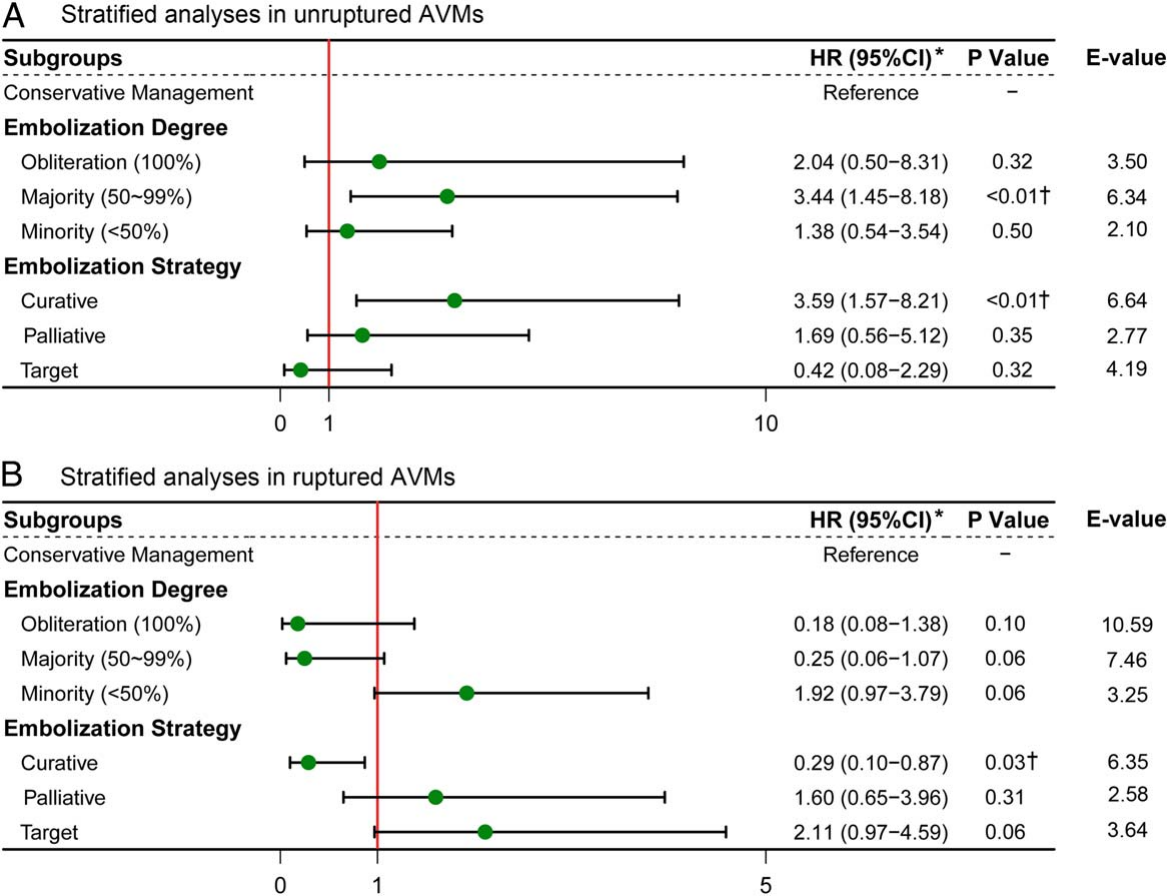
Stratified analyses based on embolization degrees and embolization strategies in unruptured and ruptured AVMs. Conservative treatment served as the control group. *HR considering the competing risk events. †Statistically significant (*P*<0.05).

In the unruptured AVMs, a complete obliteration was achieved in 17 (11.8%) cases, along with majority embolization in 57 (39.6%) cases, and minority embolization in 70 (48.6%) cases. In terms of the embolization strategy, the case number of curative embolization was 72 (50.0%), along with palliative embolization of 41 (28.5%), and target embolization of 31 (21.5%). The stratified analyses unfavored embolization, especially in patients with a majority embolized [HR, 3.44 (95% CI: 1.45–8.18)] and the curative embolization strategy [HR, 3.59 (95% CI: 1.57–8.21)]. Despite a nonstatistical significance, targeted embolization tended to prevent the primary outcome [HR, 0.42 (95% CI: 0.08–2.29)] (Fig. 3).

In the ruptured AVMs, the strategy of embolization seemed to be more aggressive: 39 cases (31.0%) with complete obliteration, 45 (35.7%) with majority embolization, and 42 (33.3%) with minority embolization. In terms of the embolization strategy, 86 (68.3%) underwent curative embolization, 27 (21.4%) received palliative embolization, and 13 (10.3%) got target embolization. In the stratified analyses, curative embolization could significantly prevent the primary outcome events (HR, 0.29 [95% CI, 0.10-0.87]) (absolute risk reduction, 0.17; number needed to treat, 6.06). The E-value indicated that unmeasured confounders required strong association with curative embolization and primary outcome by an HR of 6.35-fold to explain away the observed association. Conversely, a minor-embolized fraction [HR, 1.92 (95% CI: 0.97–3.79)], a palliative embolization [HR, 1.60 (95% CI: 0.65–3.96)], and a target embolization [HR, 2.11 (95% CI: 0.97–4.59)] might be potentially detrimental. It should be noted that 14 ischemic strokes and 1 intraoperative hemorrhagic stroke occurred during the perioperative period. Therefore, the benefits of embolization in ruptured AVMs should be interpreted with caution.

### Subgroup analyses

**Figure 4:**
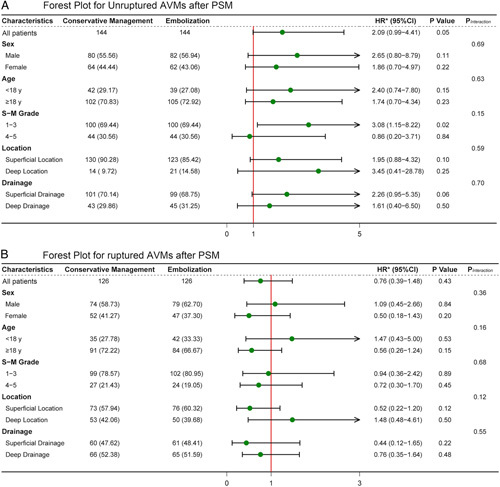
Forest plots of HRs for the primary outcome stratified by different subgroups in unruptured and ruptured AVMs. HRs for the primary outcome were stratified by sex (female and male), age at diagnosis (<18 years or ≥18 years), SM grade (1–3 or 4–5), location (superficial location or deep location), and drainage (superficial drainage or deep drainage). Conservative treatment served as the control group. *HR considering the competing risk events.

There was no significant interaction between treatment modality and sex, age, SM grade, AVM location, and drainage on the risk of the primary outcome, both in unruptured and ruptured AVMs (Fig. 4). However, embolization appeared to have a significantly higher risk of primary outcome than conservative management in the unruptured SM 1–3 AVMs [HR, 3.08 (95% CI: 1.15–8.22)].

### Secondary outcomes

In the overall cohort, the median follow-up of the secondary outcomes was 5.6 (IQR, 2.7 to 8.7) years. The mRS score at the last follow-up did not differ between conservative management and embolization in the distribution of long-term neurological outcomes (*P*=0.594) (Supplemental Figure 2, Supplemental Digital Content 2, http://links.lww.com/JS9/A574). However, conservative management was significantly better in achieving a dichotomized favorable (mRS<2) neurological outcome [RR, 1.12 (95% CI: 1.04–1.21), *P*=0.003] (Supplemental Figure 3, Supplemental Digital Content 2, http://links.lww.com/JS9/A574). In the unruptured and ruptured AVMs, the median follow-up of the secondary outcomes was 6.4 (IQR, 3.1 to 9.8) years and 4.9 (IQR, 2.1 to 7.1) years, respectively. Conservative management and embolization did not reveal significance in either the distribution of mRS score at the last follow-up (unruptured AVMs: *P*=0.746; ruptured AVMs: *P*=0.706) or the favorable neurological outcomes [unruptured AVMs: RR, 1.08 (95% CI: 0.98–1.19), *P*=0.136]; [ruptured AVMs: RR, 1.11 (95% CI: 0.96–1.28), *P*=0.153].

## Discussion

In this prospective cohort study, embolization was not superior to conservative management in improving the risk of hemorrhagic stroke or death and long-term neurological status. This study found that embolization may increase the risk of primary outcome by 109% in unruptured AVMs and decrease the risk by 24% in ruptured AVMs compared with conservative management. Furthermore, an aggressive embolization plan with subtotal obliteration might not be recommended for unruptured AVMs, and target embolization tended to be more optimal. More aggressive embolization strategies (curative embolization with subtotal or complete obliteration) could be beneficial for ruptured AVMs, rather than minor obliteration in a palliative or target embolization. The long-term neurological status was similar between these two treatment modalities.

Embolization with curative intent has been employed as a stand-alone management technique for AVMs in several case series^6,10,24^. However, most previous series only focused on immediate postoperative obliteration rates and peri-procedural complications, such as perioperative ischemic and hemorrhagic stroke and neurological deficits (temporary/permanent). Due to the heterogeneity of previously reported data, multiple confounding factors, and controlled groups of less homogeneity, the researchers cannot obtain definite conclusions regarding long-term outcomes after stand-alone embolization from previous studies^25^. Our study found a postembolization annual rupture rate of 1.64% per year and mortality of 0.42% per year, similar to a previous meta-analysis that included 14 observational cohorts (annual rupture rate of 1.70%, and mortality of 0.96% after embolization)^3^.

Although some previous case series have reported the safety and efficacy of stand-alone embolization, multiple meta-analyses of large sample sizes have failed to support the benefit of embolization over conservative management^8,19,25^, as was the case in our study. This study did not find a significant benefit of embolization over conservative management among the overall cohort, in neither the ratio of outcome events nor the survival hazards [2.07 vs. 1.57, ARD, 0.50 (95% CI: −0.34–1.34) per 100 patient-years; HR, 1.28 (95% CI: 0.81–2.04)].

Generally, the risk of subsequent hemorrhage in unruptured AVMs is much lower than that in ruptured AVMs^4^. In 2014 and 2020, the ARUBA trial suggested that medical management was superior to intervention in preventing the composite outcomes of stroke and death in unruptured AVMs^15,26^. Although, the trial has been subjected to considerable criticism^10^, the clinical-decision making is still profoundly impacted. In the ARUBA trial, 28 unruptured AVMs underwent stand-alone embolization, and up to 50% had symptomatic stroke or death during long-term follow-up, significantly higher than surgery (28.6%), SRS (24.2%), and conservative management (13.6%)^26^. Our study found similar trends as the ARUBA trial: stand-alone embolization may increase the rate of hemorrhagic stroke and death by 1.04 per 100 person-years and increase the risk by 1.09-fold compared with conservative management, especially in SM grade 1–3 unruptured AVMs (increased by 2.08-fold). These findings suggest a more specific conclusion that the stand-alone embolization for unruptured AVMs is inferior to conservative management in preventing hemorrhagic stroke and death.

For ruptured AVMs, the current clinical consensus maintains that active intervention should be considered, but the protective effect of embolization should still be determined by evidence of higher-quality^13^. In our study, it also suggested that ruptured AVMs would benefit from intervention, as the stand-alone embolization could potentially decrease the long-term risk of subsequent hemorrhagic stroke or death. However, perioperative ischemic stroke and hemorrhage during embolization should be vigilant.

With an in-depth exploration of embolization strategies, the benefits to long-term outcomes in AVMs should be clarified^18^. In terms of embolization strategy, targeted embolization of structures predisposing to rupture was often used for AVMs when complete obliteration is proposed to be impossible or risky^13,19^. Palliative embolization was proposed to improve neurologic deficits or seizures by relieving venous drainage hypertension^1^. Curative embolization tends to be appropriate for low-grade AVMs (SM grade 1–3) with simple angioarchitecture^7,27,28^. However, there is currently insufficient data to support the safety and efficacy of these embolization strategies. Many researchers suggested that the risk of subsequent hemorrhage may increase in the residual nidus after embolization^9^. Of note in our study, although stand-alone embolization was ineffective in preventing long-term hemorrhagic stroke and death in unruptured AVMs, targeted embolization might be potentially beneficial, with a 0.58-fold reduction in the risk of primary outcome. Whereas in ruptured AVMs, curative embolization showed a considerable advantage (0.71-fold reduction) in reducing the risk of hemorrhagic stroke and death. The disparity in embolization strategies may be due to different mechanisms of hemorrhage. High-risk angioarchitecture and disturbed hemodynamics may be the primary mechanism of unruptured AVMs’ subsequent hemorrhage^29^, while the ruptured AVMs’ rebleeding mechanism can be more complicated as the hematoma may stimulate inflammation and angiogenesis^30^. Failure in understanding or management of the responsible angioarchitecture for previous bleeding would not be able to prevent re-rupture. Therefore, targeted and palliative embolization may be overestimated in preventing subsequent hemorrhage of ruptured AVMs.

The complete occlusion rate of stand-alone AVM embolization has been reported to be 23.5–95%^7,9,10^, especially in low-grade AVMs (SM grade 1–3) with simple vascular structures (89.9–95%)^7,27,28^. In the present study, the complete obliteration rate of unruptured AVMs was 23.6%, and 45.3% in ruptured AVMs where curative embolization was planned, which was consistent with the previous series. However, it should not be ignored that complete obliteration does not eliminate the hemorrhagic risk, especially in unruptured AVMs, with a remaining 1.04-fold increased risk. The possible mechanism is postoperative perfusion pressure breakthrough or recanalization of the nidus^31^.

It is unclear whether stand-alone embolization can improve long-term neurological status in previous studies. Although, some previous studies reported satisfactory neurological prognosis with stand-alone embolization, these studies were not controlled with conservative management^7,9,10,27,28^. The present study compared stand-alone embolization with conservative management, and found that embolization was slightly inferior in terms of long-term neurological status. Therefore, embolization plans aimed at improving neurological status should be carefully selected, considering peri-procedural complication rate of 5.1−24.1%^8,9,24^.

Embolization is also the major treatment for AVMs of other sites, especially in pulmonary lesions^32,33^. Different liquid embolization agents and strategies are being developed and applied rapidly^32,34^. However, due to the restricted experimental design and sample size, their efficacy still remains to be determined in clinical care.

Our study had several limitations. First, the vast majority of liquid embolic agents used in the MATCH registry was Onyx, so the effect of other embolic agents on the outcomes of stand-alone embolization was not considered in the present study. Nevertheless, many previous studies have found that the type of embolic agent does not significantly affect long-term outcomes^24^. Second, AVMs in the MATCH registry who underwent stand-alone embolization all received a transarterial embolization. Therefore, the results of our study may not apply to AVMs treated with transvenous embolization which was proposed in recent years to improve the obliteration rate in selected cases^35^. Third, unlike the ARUBA trial, the primary outcomes of our study did not include ischemic and intraoperative hemorrhagic stroke. Because both of these were intraoperative complications and the focus of our study was long-term outcomes, the long-term hemorrhagic stroke or death was defined as the primary outcome. Fourth, the potential benefits of advances in endovascular techniques and microcatheter technology on clinical outcomes were not measured in our study. However, the stratified analysis may somewhat avoid such bias. Fifth, despite the fact that the PSM balanced the observed baseline characteristics, unmeasured confounders may contribute to biased estimates. E-value was used to estimate the strength of unmeasured confounders. Powerful confounders were unlikely to be present because all estimated HRs were lower than the calculated E-value. Future studies on stand-alone embolization should be more prudent with individualized consideration weighing risks versus benefits.

## Conclusions

In this prospective cohort study, our results did not support a substantial superiority of embolization over conservative management for AVMs in preventing long-term hemorrhagic stroke or death.

## Ethical approval

This study was approved by Institutional Research Ethics Committee of Beijing Tiantan Hospital (IRB approval number: KY 2020-003-01) and conducted under the guidance of the Declaration of Helsinki.

## Study funding

This work was supported by the National Key Research and Development Program of China (Grant No. 2021YFC2501101 and 2020YFC2004701 to Xiaolin Chen), Natural Science Foundation of China (grant no. 81771234 and 82071302 to Yuanli Zhao, and 82202244 to Yu Chen), Top Talent Support Program for Medical Experts Team of Wuxi Health Committee (Grant No. 202109 to Shuo Wang).

## Author contribution

Y.C.: conceptualization, methodology, formal analysis, investigation, writing – original draft, funding acquisition; H.H.: methodology, software, validation, formal analysis, investigation, visualization, data curation, writing – original draft; H.J.: resources, methodology, validation, investigation; X.M.: resources, methodology, validation, investigation; L.M.: methodology, validation, investigation; R.L.: resources, investigation; Z.L.: resources, investigation; D.Y.: resources; H.Z.: resources; K.Y.: resources; K.W.: resources; Y.Z.: resources, validation, investigation; Y.Z.: resources, validation, investigation; W.J.: validation, investigation; R.L.: resources, investigation; F.L.: resources, investigation; Q.H.: methodology; H.W.: methodology; X.Y.: methodology; S.K.: methodology; D.G.: investigation; J.P.: resources, investigation; Z.S.: resources, investigation; X.C.: resources, investigation; Z.L.: resources, investigation; J.L.: resources, investigation; J.L.: resources, investigation; S.S.: investigation; A.L.: investigation; Y.L.: conceptualization, supervision; Y.Z.: conceptualization, supervision, funding acquisition; X.C.: conceptualization, supervision, funding acquisition; S.W.: conceptualization, writing - review and editing, supervision, project administration, funding acquisition. All authors confirm that they contributed to manuscript reviews and critical revision for important intellectual content, and read and approved the final draft for submission. All authors agree to be accountable for the content of this study.

## Conflicts of interest disclosure

The authors declare no conflicts of interest relevant to this study.

## Research registration unique identifying number (UIN)

1. Name of the registry: Registry of multimodality treatment for brain AVMs in mainland China (MATCH).
2. Unique Identifying number or registration ID: ClinicalTrials.gov register, NCT 04572568.
3. Hyperlink to your specific registration (must be publicly accessible and will be checked): https://www.clinicaltrials.gov/ct2/show/NCT04572568?cond=04572568&draw=2&rank=1.

## Guarantor

Xiaolin Chen, MD, Department of Neurosurgery, Beijing Tiantan Hospital, Capital Medical University, Beijing, China. E-mail: cxl_bjtth@163.com.

## Data availability statement

All original data are available upon reasonable request to the corresponding authors.

## Supplementary Material
