# Supplementary material for: Association of embolization with long-term outcomes in brain arteriovenous malformations: a propensity score-matched analysis using nationwide multicenter prospective registry data
Source: Int J Surg. 2023 May 24;109(7):1900–9. doi: 10.1097/JS9.0000000000000341 (PMC10389468; doi:10.1097/JS9.0000000000000341)

**Context**

[Supplemental Methods. Protocol for Data Quality Management in the MATCH study 2](#_Toc122695771)

[Supplemental Table 1. Number and proportional (%) breakdown of patient characteristics at index admission for conservative management and embolization in patients lost to follow-up. 3](#_Toc122695772)

[Supplemental Table 2. Number and proportional (%) breakdown of patient characteristics at index admission for conservative management and embolization before PSM. 4](#_Toc122695773)

[Supplemental Table 3. Number and proportional (%) breakdown of patient characteristics at index admission for conservative management and embolization after PSM. 5](#_Toc122695774)

[Supplemental Figure 1. Standardized mean difference in the total, unruptured, and ruptured AVMs. 6](#_Toc122695775)

[Supplemental Figure 2: The long-term neurological outcomes in the total cohort, unruptured and ruptured AVMs. 7](#_Toc122695776)

[Supplemental Figure 3. The distribution of favorable neurofunctional status (mRS < 2) between conservative management and embolization. 8](#_Toc122695777)

# Supplemental Methods. Protocol for Data Quality Management in the MATCH study

1. Definition of variables was discussed and unified according to the terminology reporting standards or published papers before the initiation of data collection. Clinical research coordinators (CRCs) and neurosurgery residents were then trained by a cerebrovascular neurosurgeon with more than 15 years’ working experience. CRCs were responsible for demographic information and follow-up data, and neurosurgery residents for angiographic features. The two parts were blinded to each other to ensure the data collected were not biased by imaging characteristics or clinical outcomes.

2. A standard training dataset with 50 cases was used to check the consistency of data collectors. For those variables or cases with significant interobserver variation, the consensus was reached by either modifying the confusing definitions or retraining the data collectors. Only when the consistency reached 90% can the CRC or the resident be allowed to extract information independently.

3. While recording data, one could ask for help about unsure cases in a discussion group with cerebrovascular neurosurgeons in it or mark these cases and discuss them in weekly meetings.

4. The group leader with more than five years’ working experience randomly spot-checks these data biweekly. Investigators would receive training again if their data were of low quality, and these data would be recollected by other investigators.

# Supplemental Table 1. Number and proportional (%) breakdown of patient characteristics at index admission for conservative management and embolization in patients lost to follow-up.

|  | Unruptured AVM  (n=25) | | Ruptured AVM  (n=56) | |
| --- | --- | --- | --- | --- |
|  | Conservative Management | Embolization | Conservative Management | Embolization |
| No. of patients | 8 | 17 | 11 | 45 |
| Sex (female) | 2 (8.0) | 6 (24.0) | 6 (10.7) | 19 (33.9) |
| Age at diagnosis, mean (SD) | 32.4 (15.6) | 35.9 (14.5) | 32.1 (11.0) | 26.5 (13.6) |
| Admission mRS, median (IQR) | 1 (0, 1) | 1 (1, 1) | 1 (1, 2) | 1 (0, 2) |
| Seizure | 0 (0.0) | 3 (12.0) | 1 (1.8) | 2 (3.6) |
| Neurological deficiency | 2 (8.0) | 2 (8.0) | 1 (1.8) | 6 (10.7) |
| Location (supratentorial) | 7 (28.0) | 16 (64.0) | 8 (14.3) | 35 (62.5) |
| Spetzler-Martin grade (I–III) | 5 (20.0) | 14 (56.0) | 8 (14.3) | 35 (62.5) |
| Ventricular system involvement | 2 (8.0) | 4 (16.0) | 6 (10.7) | 30 (53.6) |
| Size (<3cm) | 2 (8.0) | 6 (24.0) | 5 (8.9) | 22 (39.3) |
| Eloquent region | 5 (20.0) | 4 (16.0) | 8 (14.3) | 28 (50.0) |
| Feeding artery dilation | 5 (20.0) | 13 (52.0) | 4 (7.1) | 16 (28.6) |
| Single feeder | 0 (0.0) | 1 (4.0) | 3 (5.4) | 20 (35.7) |
| Multiple source supply | 2 (8.0) | 8 (32.0) | 2 (3.6) | 9 (16.1) |
| ACA supply | 2 (8.0) | 8 (32.0) | 5 (8.9) | 11 (19.6) |
| MCA supply | 6 (24.0) | 12 (48.0) | 5 (8.9) | 19 (33.9) |
| PCirA supply | 2 (8.0) | 7 (28.0) | 3 (5.4) | 25 (44.6) |
| Perforating artery | 2 (8.0) | 5 (20.0) | 2 (3.6) | 19 (33.9) |
| Flow-related aneurysm | 0 (0.0) | 6 (24.0) | 2 (3.6) | 9 (16.1) |
| Diffuse nidus | 2 (8.0) | 5 (20.0) | 4 (7.1) | 19 (33.9) |
| Exclusive deep drainage | 1 (4.0) | 1 (4.0) | 4 (7.1) | 14 (25.0) |
| Any deep drainage | 4 (16.0) | 4 (16.0) | 5 (8.9) | 22 (39.3) |
| Single drainage vein | 4 (16.0) | 6 (24.0) | 5 (8.9) | 27 (48.2) |
| Draining vein stenosis | 1 (4.0) | 0 (0.0) | 3 (5.4) | 9 (16.1) |
| Venous aneurysm | 6 (24.0) | 7 (28.0) | 3 (5.4) | 3 (5.4) |

# Supplemental Table 2. Number and proportional (%) breakdown of patient characteristics at index admission for conservative management and embolization before PSM.

| Characteristics | Total Cohort (%) | | |  | Unruptured AVMs (%) | | |  | Ruptured AVMs (%) | | |
| --- | --- | --- | --- | --- | --- | --- | --- | --- | --- | --- | --- |
|  | Conservative Management | Embolization | *p* value |  | Conservative Management | Embolization | *p* value |  | Conservative Management | Embolization | *p* value |
| No. of patients | 448 | 377 |  |  | 297 | 156 |  |  | 151 | 221 |  |
| Sex (female) | 173 (38.6) | 149 (39.5) | 0.846 |  | 111 (37.4) | 66 (42.3) | 0.357 |  | 62 (41.1) | 83 (37.6) | 0.567 |
| Age at diagnosis, mean (SD) | 28.3 (15.8) | 27.9 (15.0) | 0.697 |  | 28.6 (15.7) | 27.9 (13.7) | 0.655 |  | 27.8 (15.9) | 27.9 (15.9) | 0.962 |
| Admission mRS, median (IQR) | 1.0 (1.0, 1.0) | 1.0 (0.0, 1.0) | 0.234 |  | 1.0 (1.0, 1.0) | 1.0 (1.0, 1.0) | 0.392 |  | 1.0 (1.0, 2.0) | 1.0 (0.0, 2.0) | 0.039* |
| Seizure | 119 (26.6) | 79 (21.0) | 0.072 |  | 107 (36.0) | 58 (37.2) | 0.889 |  | 12 (7.9) | 21 (9.5) | 0.740 |
| Neurological deficiency | 107 (23.9) | 79 (21.0) | 0.358 |  | 76 (25.6) | 40 (25.6) | >0.999 |  | 31 (20.5) | 39 (17.6) | 0.573 |
| Location (supratentorial) | 370 (82.6) | 311 (82.5) | 0.971 |  | 251 (84.5) | 141 (90.4) | 0.082 |  | 119 (78.8) | 170 (76.9) | 0.668 |
| Spetzler-Martin grade (I–III) | 301 (67.2) | 290 (76.9) | 0.002* |  | 187 (63.0) | 111 (71.2) | 0.081 |  | 114 (75.5) | 179 (81.0) | 0.203 |
| Ventricular system involvement | 237 (52.9) | 184 (48.8) | 0.270 |  | 125 (42.1) | 51 (32.7) | 0.065 |  | 112 (74.2) | 133 (60.2) | 0.007* |
| Size (<3cm) | 150 (33.5) | 160 (42.4) | 0.010* |  | 66 (22.2) | 27 (17.3) | 0.268 |  | 84 (55.6) | 133 (60.2) | 0.443 |
| Eloquent region | 287 (64.1) | 218 (57.8) | 0.078 |  | 194 (65.3) | 91 (58.3) | 0.174 |  | 93 (61.6) | 127 (57.5) | 0.492 |
| Feeding artery dilation | 252 (56.2) | 220 (58.4) | 0.590 |  | 199 (67.0) | 127 (81.4) | 0.002* |  | 53 (35.1) | 93 (42.1) | 0.213 |
| Single feeder | 97 (21.7) | 93 (24.7) | 0.346 |  | 31 (10.4) | 20 (12.8) | 0.545 |  | 66 (43.7) | 73 (33.0) | 0.048* |
| Multiple source supply | 179 (40.0) | 120 (31.8) | 0.019* |  | 141 (47.5) | 70 (44.9) | 0.668 |  | 38 (25.2) | 50 (22.6) | 0.658 |
| ACA supply | 170 (37.9) | 122 (32.4) | 0.110 |  | 123 (41.4) | 60 (38.5) | 0.612 |  | 47 (31.1) | 62 (28.1) | 0.601 |
| MCA supply | 297 (66.3) | 215 (57.0) | 0.008* |  | 220 (74.1) | 110 (70.5) | 0.485 |  | 77 (51.0) | 105 (47.5) | 0.579 |
| PCirA supply | 204 (45.5) | 180 (47.7) | 0.573 |  | 134 (45.1) | 68 (43.6) | 0.833 |  | 70 (46.4) | 112 (50.7) | 0.476 |
| Perforating artery | 228 (50.9) | 147 (39.0) | 0.001* |  | 140 (47.1) | 50 (32.1) | 0.003* |  | 88 (58.3) | 97 (43.9) | 0.009* |
| Flow-related aneurysm | 88 (19.6) | 86 (22.8) | 0.305 |  | 55 (18.5) | 33 (21.2) | 0.583 |  | 33 (21.9) | 53 (24.0) | 0.724 |
| Diffuse nidus | 190 (42.4) | 131 (34.7) | 0.029* |  | 108 (36.4) | 40 (25.6) | 0.027* |  | 82 (54.3) | 91 (41.2) | 0.017* |
| Exclusive deep drainage | 93 (20.8) | 81 (21.5) | 0.866 |  | 32 (10.8) | 10 (6.4) | 0.177 |  | 61 (40.4) | 71 (32.1) | 0.127 |
| Any deep drainage | 197 (44.0) | 152 (40.3) | 0.323 |  | 113 (38.0) | 46 (29.5) | 0.087 |  | 84 (55.6) | 106 (48.0) | 0.178 |
| Single drainage vein | 206 (46.0) | 185 (49.1) | 0.415 |  | 93 (31.3) | 46 (29.5) | 0.769 |  | 113 (74.8) | 139 (62.9) | 0.021* |
| Draining vein stenosis | 69 (15.4) | 70 (18.6) | 0.264 |  | 35 (11.8) | 18 (11.5) | >0.999 |  | 34 (22.5) | 52 (23.5) | 0.918 |
| Venous aneurysm | 112 (25.0) | 75 (19.9) | 0.097 |  | 101 (34.0) | 57 (36.5) | 0.665 |  | 11 (7.3) | 18 (8.1) | 0.915 |

ACA, Anterior Cerebral Artery; AVM, Arteriovenous Malformation; IQR, Interquartile Range; MCA, Middle Cerebral Artery; mRS, modified Rankin Scale; PCirA, Posterior Circulation Artery; PSM, Propensity Score Matching; SD, Standardized Difference

*Statistical significance (*p* < 0.05)

# Supplemental Table 3. Number and proportional (%) breakdown of patient characteristics at index admission for conservative management and embolization after PSM.

| Characteristics | Total Cohort (%) | | |  | Unruptured AVMs (%) | | |  | Ruptured AVMs (%) | | |
| --- | --- | --- | --- | --- | --- | --- | --- | --- | --- | --- | --- |
|  | Conservative Management | Embolization | P value |  | Conservative Management | Embolization | P value |  | Conservative Management | Embolization | P value |
| No. of patients | 311 | 311 |  |  | 144 | 144 |  |  | 126 | 126 |  |
| Sex (female) | 124 (39.9) | 119 (38.3) | 0.742 |  | 64 (44.4) | 62 (43.1) | 0.905 |  | 52 (41.3) | 47 (37.3) | 0.606 |
| Age at diagnosis, mean (SD) | 28.5 (15.7) | 27.7 (15.2) | 0.487 |  | 28.2 (15.6) | 28.1 (13.6) | 0.962 |  | 28.2 (15.3) | 28.0 (16.4) | 0.909 |
| Admission mRS, median (IQR) | 1.0 (1.0, 1.0) | 1.0 (1.0, 2.0) | 0.655 |  | 1.0 (1.0, 1.0) | 1.0 (1.0, 1.0) | 0.690 |  | 1.0 (1.0, 2.0) | 1.0 (1.0, 2.0) | 0.573 |
| Seizure | 69 (22.2) | 74 (23.8) | 0.703 |  | 56 (38.9) | 54 (37.5) | 0.903 |  | 10 (7.9) | 9 (7.1) | >0.999 |
| Neurological deficiency | 69 (22.2) | 70 (22.5) | >0.999 |  | 31 (21.5) | 38 (26.4) | 0.407 |  | 27 (21.4) | 27 (21.4) | >0.999 |
| Location (supratentorial) | 264 (84.9) | 254 (81.7) | 0.283 |  | 133 (92.4) | 129 (89.6) | 0.411 |  | 101 (80.2) | 100 (79.4) | 0.875 |
| Spetzler-Martin grade (I-III) | 227 (73.0) | 234 (75.2) | 0.522 |  | 100 (69.4) | 100 (69.4) | >0.999 |  | 99 (78.6) | 102 (81.0) | 0.638 |
| Ventricular system involvement | 156 (50.2) | 157 (50.5) | >0.999 |  | 49 (34.0) | 50 (34.7) | >0.999 |  | 89 (70.6) | 90 (71.4) | >0.999 |
| Size (<3cm) | 116 (37.3) | 119 (38.3) | 0.869 |  | 23 (16.0) | 25 (17.4) | 0.874 |  | 73 (57.9) | 74 (58.7) | >0.999 |
| Eloquent region | 184 (59.2) | 190 (61.1) | 0.682 |  | 86 (59.7) | 87 (60.4) | >0.999 |  | 74 (58.7) | 75 (59.5) | >0.999 |
| Feeding artery dilation | 188 (60.5) | 179 (57.6) | 0.514 |  | 114 (79.2) | 116 (80.6) | 0.883 |  | 43 (34.1) | 45 (35.7) | 0.895 |
| Single feeder | 72 (23.2) | 67 (21.5) | 0.700 |  | 14 (9.7) | 17 (11.8) | 0.704 |  | 54 (42.9) | 54 (42.9) | >0.999 |
| Multiple source supply | 116 (37.3) | 107 (34.4) | 0.504 |  | 66 (45.8) | 67 (46.5) | >0.999 |  | 29 (23.0) | 29 (23.0) | >0.999 |
| ACA supply | 113 (36.3) | 104 (33.4) | 0.501 |  | 60 (41.7) | 57 (39.6) | 0.810 |  | 40 (31.7) | 40 (31.7) | >0.999 |
| MCA supply | 200 (64.3) | 192 (61.7) | 0.561 |  | 105 (72.9) | 104 (72.2) | >0.999 |  | 62 (49.2) | 63 (50.0) | >0.999 |
| PCirA supply | 134 (43.1) | 142 (45.7) | 0.572 |  | 58 (40.3) | 61 (42.4) | 0.811 |  | 57 (45.2) | 56 (44.4) | >0.999 |
| Perforating artery | 130 (41.8) | 132 (42.4) | 0.935 |  | 49 (34.0) | 48 (33.3) | >0.999 |  | 68 (54.0) | 62 (49.2) | 0.529 |
| Flow-related aneurysm | 72 (23.2) | 73 (23.5) | >0.999 |  | 34 (23.6) | 30 (20.8) | 0.671 |  | 26 (20.6) | 26 (20.6) | >0.999 |
| Diffuse nidus | 119 (38.3) | 118 (37.9) | >0.999 |  | 41 (28.5) | 38 (26.4) | 0.792 |  | 65 (51.6) | 60 (47.6) | 0.614 |
| Exclusive deep drainage | 64 (20.6) | 65 (20.9) | >0.999 |  | 7 (4.9) | 10 (6.9) | 0.617 |  | 47 (37.3) | 46 (36.5) | >0.999 |
| Any deep drainage | 126 (40.5) | 125 (40.2) | >0.999 |  | 43 (29.9) | 45 (31.2) | 0.898 |  | 66 (52.4) | 65 (51.6) | >0.999 |
| Single drainage vein | 147 (47.3) | 150 (48.2) | 0.872 |  | 44 (30.6) | 41 (28.5) | 0.796 |  | 93 (73.8) | 95 (75.4) | 0.885 |
| Draining vein stenosis | 48 (15.4) | 50 (16.1) | 0.912 |  | 16 (11.1) | 15 (10.4) | >0.999 |  | 28 (22.2) | 26 (20.6) | 0.878 |
| Venous aneurysm | 67 (21.5) | 62 (19.9) | 0.692 |  | 51 (35.4) | 56 (38.9) | 0.626 |  | 10 (7.9) | 8 (6.3) | 0.807 |

ACA, Anterior Cerebral Artery; AVM, Arteriovenous Malformation; IQR, Interquartile Range; MCA, Middle Cerebral Artery; mRS, modified Rankin Scale; PCirA, Posterior Circulation Artery; PSM, Propensity Score Matching; SD, Standardized Difference

*Statistical significance (P<0.05)

# Supplemental Figure 1. Standardized mean difference in the total, unruptured, and ruptured AVMs. ACA, Anterior Cerebral Artery; MCA, Middle Cerebral Artery; mRS, modified Rankin Scale; PCirA, Posterior Circulation Artery


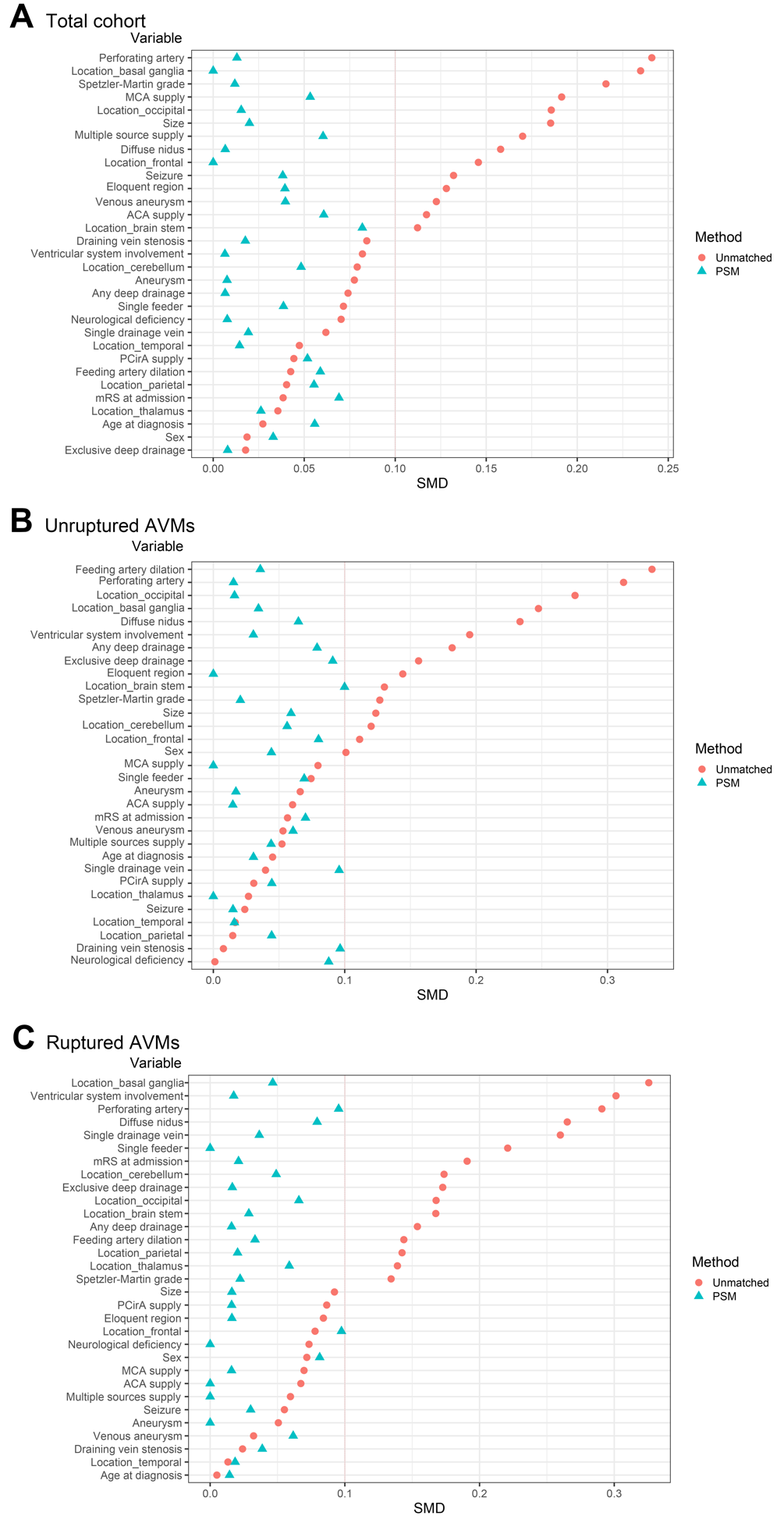


# Supplemental Figure 2: The long-term neurological outcomes in the total cohort, unruptured and ruptured AVMs. P values are shown before and after adjusting for competing risk events. AVM, arteriovenous malformation; mRS, modified Rankin Scale; PSM, propensity score matching

**
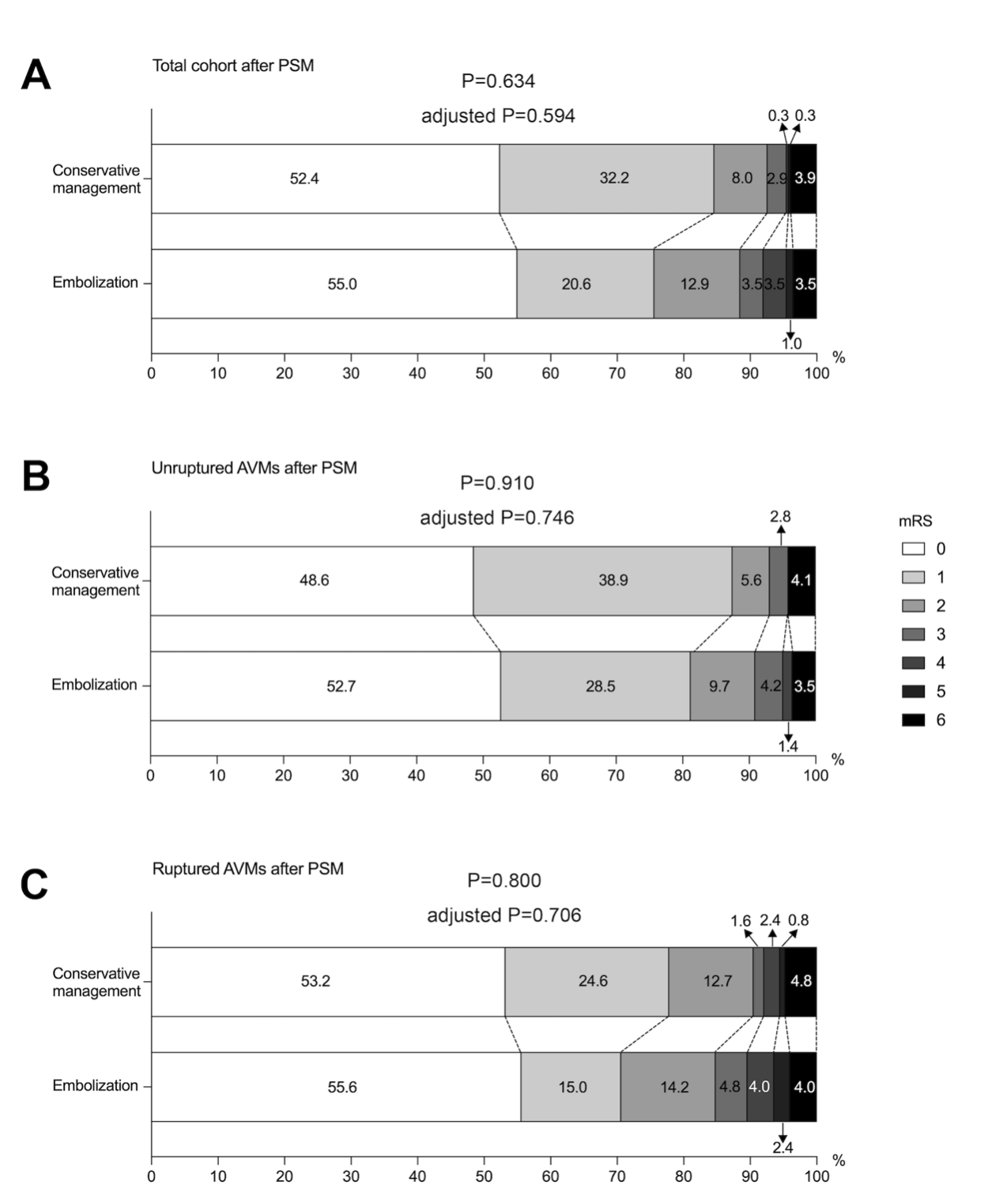
**

# Supplemental Figure 3. The distribution of favorable neurofunctional status (mRS < 2) between conservative management and embolization. P values are shown before and after adjusting for competing risk events. AVM, arteriovenous malformation; mRS, modified Rankin Scale


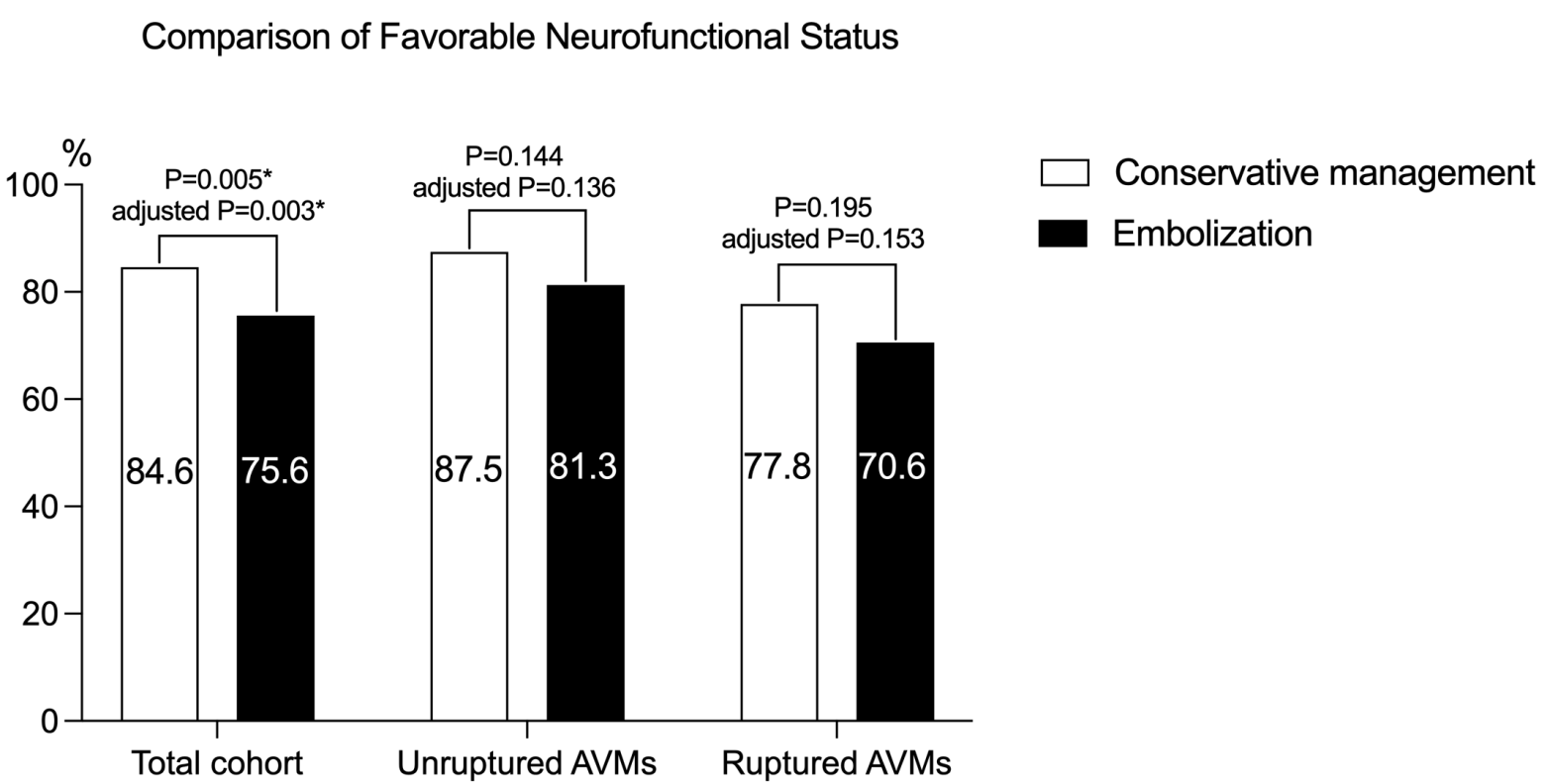

Supplement: Supplementary file 2 [file js9-109-1900-s002.docx]
